# The Incidence and Risk Factors of Associated Acute Myocardial Infarction (AMI) in Acute Cerebral Ischemic (ACI) Events in the United States

**DOI:** 10.1371/journal.pone.0105785

**Published:** 2014-08-28

**Authors:** Ali Seifi, Kevin Carr, Mitchell Maltenfort, Michael Moussouttas, Lee Birnbaum, Augusto Parra, Owoicho Adogwa, Rodney Bell, Fred Rincon

**Affiliations:** 1 Department of Neurological Surgery, University of Texas Health Sciences Center, San Antonio, Texas, United States of America; 2 Rothman Institute, Thomas Jefferson University, Philadelphia, Pennsylvania, United States of America; 3 Division of Neuro Critical Care, Capital Institute for Neurosciences, Trenton, New Jersey, United States of America; 4 Department of Neurology, University of Texas Health Sciences Center, San Antonio, Texas, United States of America; 5 Division of Neurosurgery, Department of Surgery, Duke University Medical Center, Durham, North Carolina, United States of America; 6 Department of Neurosurgery, Thomas Jefferson University, Philadelphia, Pennsylvania, United States of America; 7 Department of Neurology, Thomas Jefferson University, Philadelphia, Pennsylvania, United States of America; Hospital de Clínicas de Porto Alegre, Brazil

## Abstract

**Objectives:**

To determine the association between myocardial infarction (AMI) and clinical outcome in patients with primary admissions diagnosis of acute cerebral ischemia (ACI) in the US.

**Methods:**

Data from Nationwide Inpatient Sample (NIS) was queried from 2002–2011 for inpatient admissions of patients with a primary diagnosis of ACI with and without AMI using International Classification of Diseases, Ninth Revision, Clinical Modification coding (ICD-9). A multivariate stepwise regression analysis was performed to assess the correlation between identifiable risk factors and clinical outcomes.

**Results:**

During 10 years the NIS recorded 886,094 ACI admissions with 17,526 diagnoses of AMI (1.98%). The overall cumulative mortality of cohort was 5.65%. In-hospital mortality was associated with AMI (aOR 3.68; 95% CI 3.49–3.88, p≤0.0001), rTPA administration (aOR 2.39 CI, 2.11–2.71, p<0.0001), older age (aOR 1.03, 95% CI, 1.03–1.03, P<0.0001) and women (aOR 1.06, 95% CI 1.03–1.08, P<0.0001). Overall, mortality risk declined over the course of study; from 20.46% in 2002 to 11.8% in 2011 (OR 0.96, 95% CI 0.95–0.96, P<0.0001). Survival analysis demonstrated divergence between the AMI and non-AMI sub-groups over the course of study (log-rank p<0.0001).

**Conclusion:**

Our study demonstrates that although the prevalence of AMI in patients hospitalized with primary diagnosis of ACI is low, it negatively impacts survival. Considering the high clinical burden of AMI on mortality of ACI patients, a high quality monitoring in the event of cardiac events should be maintained in this patient cohort. Whether prompt diagnosis and treatment of associated cardiovascular diseases may improve outcome, deserves further study.

## Introduction

Acute cerebral ischemia (ACI) is a crippling medical condition worldwide and the second leading cause of death. [1], [2] In the United States it is also associated with high morbidity, mortality and associated health care resource utilization. [3] Studies from the Center for diseases control (CDC) lists it among the largest cause of mortality in the United States accounting for approximately 130 000 deaths annually and is the most morbid of cardiovascular disease states. [4], [5] Economically, the effects on the US economy are staggering with over $50B USD annually in direct and indirect costs. [3] While the incidence of ACI has declined over the past three decades, the associated morbidity remains high despite improved diagnostic tools and therapies [5], [6].

Patients with primary cerebrovascular disease often have systemic cardiovascular diseases such as coronary artery disease (CAD), diabetes, and peripheral vascular disease (PVD). Similarly the hospitalized patient with primary diagnoses of ACI can present with a constellation of underlying comorbid cardiovascular conditions that increase morbidity and mortality during inpatient admission. Collectively, the death rate attributable to cardiovascular diseases was estimated at 235.5 per 100 000 in 2010, [5] with diseases of the heart accounting for 24.2% of deaths in the United States. [7] When compared to non-hospitalized patients, hospitalized patients with non-cardiac primary admissions harbor a higher mortality risk [8].

Early thrombolytic therapy for coronary reperfusion after acute ischemia improves mortality rates in hospitalized patients. [9], [10], [11] In the cohort of patients hospitalized with ACI and shown to have cardiovascular infarction requiring chemical reperfusion, little is known about inpatient morbidity and mortality. Cerebral ischemia and CAD are epidemiologically and biologically closely related diseases. [12] In patients with AMI, stroke risk is markedly increased especially in the acute interval. Likewise, in patients surviving ACI, other manifestations of cardiovascular disease, particularly CAD, are some of the main causes of long-term mortality. [13] However, in the patients with a primary diagnosis of ACI reliable estimates of the absolute risk of associated AMI, the effect of AMI on mortality, and other factors that are associated with mortality have been lacking. The purpose of this study is to address these limitations in the existing literature by studying a large administrative cohort. We hypothesized that in patients hospitalized with a primary diagnosis of ACI, developing concomitant AMI increases odds of mortality and decreases survival.

## Methods

### Data source

The Nationwide Inpatient Sample represents approximately 20% of all hospitalizations across the United States and is anonymized and de-identified. Demographic information (age, sex, racial background, geographic location, and marital status), primary payer, and disposition at discharge are abstracted using International Classification of Disease, 9th Revision, Clinical Modification (ICD-9-CM) criteria. From 2002 onward, a set of 29 comorbidity fields are available for chronic conditions such as AIDS, diabetes, and liver disease. The Institutional Review Board of Thomas Jefferson University Hospital, Philadelphia exempted this analysis from full review.

### Sample, Definitions and Hospitalization Data

Admissions of patients with ACI were identified by querying of the database between 2002 and 2011 using the ICD-9 codes 430–438, **(** **Table 1** **)**. Goldstein etal, [14] demonstrated validity of these code for the identification of ACI. Patients were secondarily cross-matched for inpatient administration of thrombolytic by ICD-9 code 99.10 and for the presence of AMI by ICD-9 codes 410.0–410.9; both previously validated instruments in prior studies [14], [15], [16].

**Table 1:** ICD codes.

ICD-9 Codes	Definition
*Acute Cerebral Ischemic events*	
430	Subarachnoid hemorrhage
431	Intracerebral hemorrhage
432	Other and unspecified hemorrhage
433	Occlusion and stenosis of pre-cerebral arteries
434	Occlusion of cerebral arteries
435	Transient cerebral ischemia
436	Acute, but ill-defined, cerebrovascular disease
437	Other and ill-defined cerebrovascular disease
438	Late effects of cerebrovascular disease
*Injurious cardiac events*	
410.0	Acute myocardial infarction of anterolateral wall
410.1	Acute myocardial infarction of other anterior wall
410.2	Acute myocardial infarction of inferolateral wall
410.3	Acute myocardial infarction of infer posterior wall
410.4	Acute myocardial infarction of other inferior wall
410.5	Acute myocardial infarction of other lateral wall
410.6	True posterior wall infarction
410.7	Sub-endocardial infarction
410.8	Acute myocardial infarction of other specified sites
410.9	Acute myocardial infarction of unspecified site
*Administration of IV rTPA*	
99.10	Injection of infusion of thrombolytic agent

The NIS hospitalization data on adult patients (18 years of age or older) with ACI and AMI were compiled. The 2 cohorts (ACI patients who received IV rTPA and those who did not receive IV rTPA) were compared relative to two primary end points:1) in-hospital mortality and 2) the occurrence of AMI.

Based on AHRQ data was collected on co-morbidities that were identified as coexisting medical conditions, not directly related to the principal diagnosis or the main reason for admission, and likely to have originated prior to the hospital stay. Co-morbidities are identified using ICD-9-CM diagnoses and the Diagnosis Related Group (DRG) on the discharge date. Co-morbidities assessed were based on results from similar cohort studies and included neurological disorders, congestive heart failure (CHF), metastatic cancer, chronic anemia, fluid and electrolyte disorders, coagulopathy, renal failure, paralysis, pulmonary circulation disorders, alcohol abuse, cardiac valvular disease, drug abuse, peripheral vascular disorders, hypothyroidism, obesity, psychosis, depression and hypertension.

### Statistical analysis

For days to primary outcome (in-hospital mortality) analysis, Kaplan-Meier survival estimates and log-rank tests were used to compare the AMI and non-AMI groups. Prevalence proportions were calculated over the study period, and multivariate logistic models were fitted to assess the impact for AMI on in-hospital mortality and for IV rTPA on AMI.

The multivariate analysis proceeded in two stages. In the first stage, significant risk factors were identified from the candidate variables. Variables of statistical significant (p<0.05) were considered potential candidates for multivariate analysis. Given the dichotomous characteristic of the primary outcome of interest (in-hospital mortality), logistic regression modeling was used to calculate odds ratios (OR) and 95% CI. In all multivariate analyses, all factors of interest were included, and parsimonious models were found by backward stepwise elimination. The analysis was conducted using the RMS package in the R programming language for statistical computing (R Foundation for Statistical Computing, Vienna Austria), both available under the GNU Public License (http://cran.r-project.org).

## Results

During the ten years study period, the NIS reported 886,094 admissions with a primary diagnosis of ACI of which 17,526 were reported with AMI during the same inpatient admission (1.98%). There were 26,693 (3.01%) patients who received IV rTPA during hospitalization.

### Demographics

Patients admitted with a primary diagnosis of ACI were on average 71.71±14.23 (mean ± SD) years old and those with AMI were older than those without, (75.46±12.97 vs 71.63±14.25). Overall, the majority of patients with ACI (54.0%) and AMI (56.24%) were females. Geographically, most diagnoses of AMI were reported in the southern states (38.05%), urban private hospitals, (45.67%) and large size hospitals (64.14%), **Table 2**.

**Table 2:** Description of ACI patients associated with AMI.

	No AMI	AMI
	Overall, N = 868568 (Column %)	Overall, N = 17525 (Column %)
Age (mean ± SD)	71.63±14.25	75.46±12.97
*Sex*		
Female	469584 (54.06)	9857(56.24%)
*Race*		
White	483261 (55.64)	10220 (58.31)
Black	110958 (12.77)	2076 (11.85)
Hispanic	50334 (5.80)	989 (5.64)
Asian	17 316 (1.99)	376 (2.15)
Native American	2 940 (0.34)	60 (0.34)
Other	16323 (1.88)	358 (2.04)
Unspecified	187436 (21.58)	3447 (19.67)
*Hospital Size*		
small	107062 (12.33)	1885 (10.75)
medium	213667 (24.60)	4401 (25.11)
large	547838 (63.07)	11240 (64.14)
*Center type*		
urban academic	390155 (44.92)	7258 (41.41)
rural	129776 (14.94)	2264 (12.92)
urban private	348637 (40.14)	8003 (45.67)
*Geography*		
Northeast	152047 (17.51)	3597 (20.52)
Midwest	197569 (22.75)	4007 (22.86)
South	362394 (41.72)	6669 (38.05)
West	156558 (18.02)	3253 (18.56)

### Morbidity/Mortality

The total mortality was 5.65% for all patients admitted with a primary diagnosis of ACI. In hospital mortality for those patients who had associated AMI was 22.65% and for those who received IV rTPA, 6.16%. Mortality was higher in patients with reported AMI who were treated with IV rTPA (24.64%).

**Figure 1:**
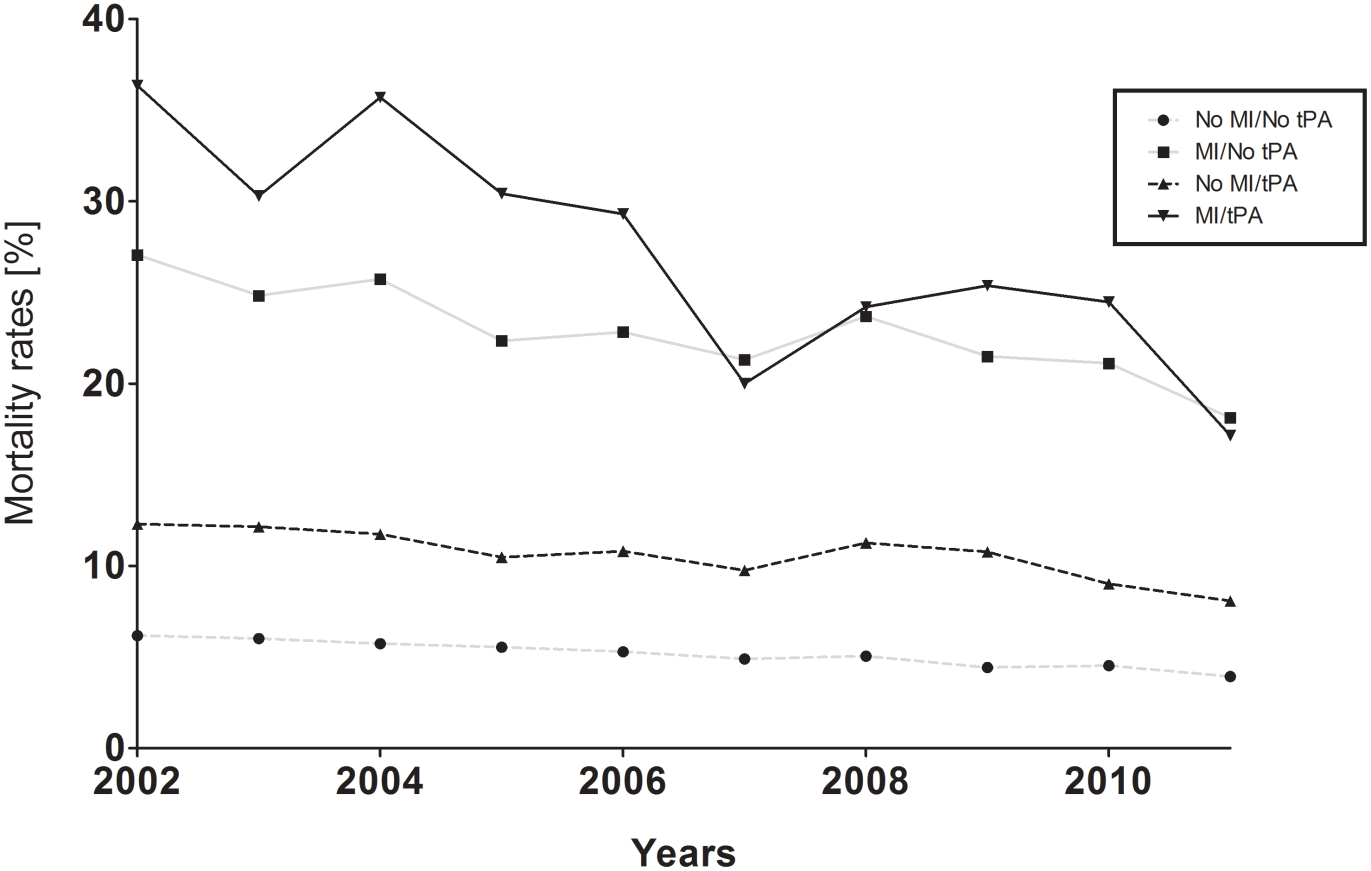
Annual mortality: Inpatients admitted with ACI.

In multivariate regression analysis, patients who were found to have AMI (aOR 3.68 CI, 3.49–3.88, p<0.0001) and those who received rTPA (aOR 2.39 CI, 2.11–2.71, p<0.0001) had statistically higher associations with inpatient mortality estimates. Nonetheless, in assessing annual estimates of rTPA administration (rTPA*Year), its association to mortality was slightly decreased (aOR 0.98, 95% CI0.95–0.98, P<0.0001). Similarly, older age (aOR 1.03, 95% CI, 1.03–1.03, P<0.0001) and female gender (aOR 1.06, 95% CI 1.03–1.08, P<0.0001), were independently associated with inpatient mortality. Overall, mortality risk declined significantly over the course of study; from 20.46% in 2002 to 11.8% in 2011 (OR 0.96, 95% CI 0.95–0.96, P<0.0001), **Figure 1**.

**Figure 2:**
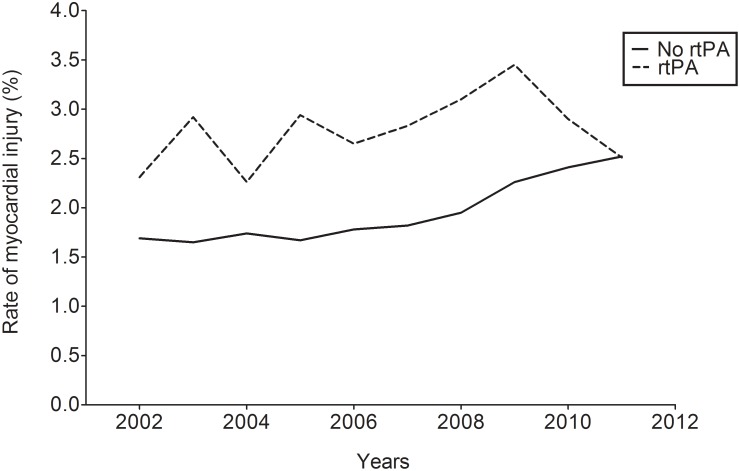
Increased risk of associated AMI in patients treated with IV rTPA.

Reports of AMI increased from 1.95% to 2.79% during the decade of study. This increase was more constant for those who did not receive rTPA compare to those who did, **Figure 2**. In our multivariate regression analysis, the administration of rTPA was associated with a diagnosis of AMI (aOR 1.91, 95% CI 1.51–2.42, P<0.0001). Patients who received IV rTPA had a significantly greater likelihood of a concomitantly reported AMI during hospital admission (aOR 1.91, 95% CI, 1.51–2.42, p<0.0001) and patients with AMI were more likely than to have multiple co-morbidities, **Table 3**.

**Table 3:** Major comorbidities associated with AMI.

Comorbidity	AMI	No AMI
	N (%)	N (%)
Anemia	89 552 (10.31%)	2 650 (15.12%)
Congestive heart failure	115 645 (13.31%)	6 103 (34.82%)
Chronic lung disease	125 336 (14.43%)	2 990 (17.06%)
Depression	72 903 (8.39%)	1 065 (6.07%)
Diabetes Mellitus	233 078 (26.83%)	4 393 (25.06%)
Hypertension	656 969 (75.64%)	11 996 (68.45%)
Electrolyte abnormality	154 625 (17.8%)	5 472 (31.22%)
Obesity	50 825 (5.85%)	728 (4.15%)
Peripheral vascular disease	69 285 (7.98%)	1 01 (9.71%)
Renal failure	78 992 (9.09%)	2 928 (16.70%)

There was no significant association between female gender and a diagnosis of AMI (aOR 1.03, 95% CI 0.99–1.07, P = 0.116), and African-Americans were slightly less likely to develop AMI (aOR 0.9, 95% CI, 0.89–0.97, P≤0.0001). There was a small but statistically significant correlation between increasing age and associated report of AMI (aOR 1.01, 95% CI, 1.01–1.02, p<0.0001).

In our multivariate regression analysis, the concomitant diagnosis of congestive heart failure, acute blood loss, metastatic disease, concomitant neurological disease, coagulopathy, renal failure and paralysis were associated with significantly increased odds for developing a reported AMI, **Table 4**. Being African-American was slightly protective to be associated with AMI in multivariate analysis (aOR 0.85, 95% CI 0.82–0.87, P≤0.0001).

**Table 4:** Multivariate regression analysis predicting odds of having associated AMI.

Comorbidities	Odds Ratio	95% Confidence Interval	P value
Congestive Heart Failure	2.79	2.68–2.89	<0.0001
Neurological pathology	2.34	2.03–2.69	<0.0001
Metastatic disease	2.21	2.00–2.45	<0.0001
rTPA administration	1.91	1.51–2.42	<0.0001
Acute blood loss	1.77	1.51–2.07	<0.0001
Coagulopathy	1.7	1.57–1.84	<0.0001
Electrolyte abnormality	1.68	1.62–1.74	<0.0001
Paralysis	1.56	1.46–1.68	<0.0001
Renal failure	1.43	1.36–1.50	<0.0001
Pathologic weight loss	1.39	1.29–1.50	<0.0001
Substance Abuse	1.34	1.16–1.54	<0.0001
Valvular disease	1.21	1.15–1.27	<0.0001
Tumor	1.19	1.08–1.32	0.0007
Alcohol use	1.16	1.05–1.27	0.0034
Anemia	1.12	1.06–1.17	<0.0001
Year	1.05	1.04–1.05	<0.0001
rTPA per Year	0.91	0.88–0.94	<0.0001
Obesity	0.85	0.78–0.92	<0.0001
Hypothyroidism	0.84	0.79–0.89	<0.0001
Depression	0.73	0.68–0.79	<0.0001
Hypertension	0.72	0.70–0.75	<0.0001

### Survival Analysis

**Figure 3:**
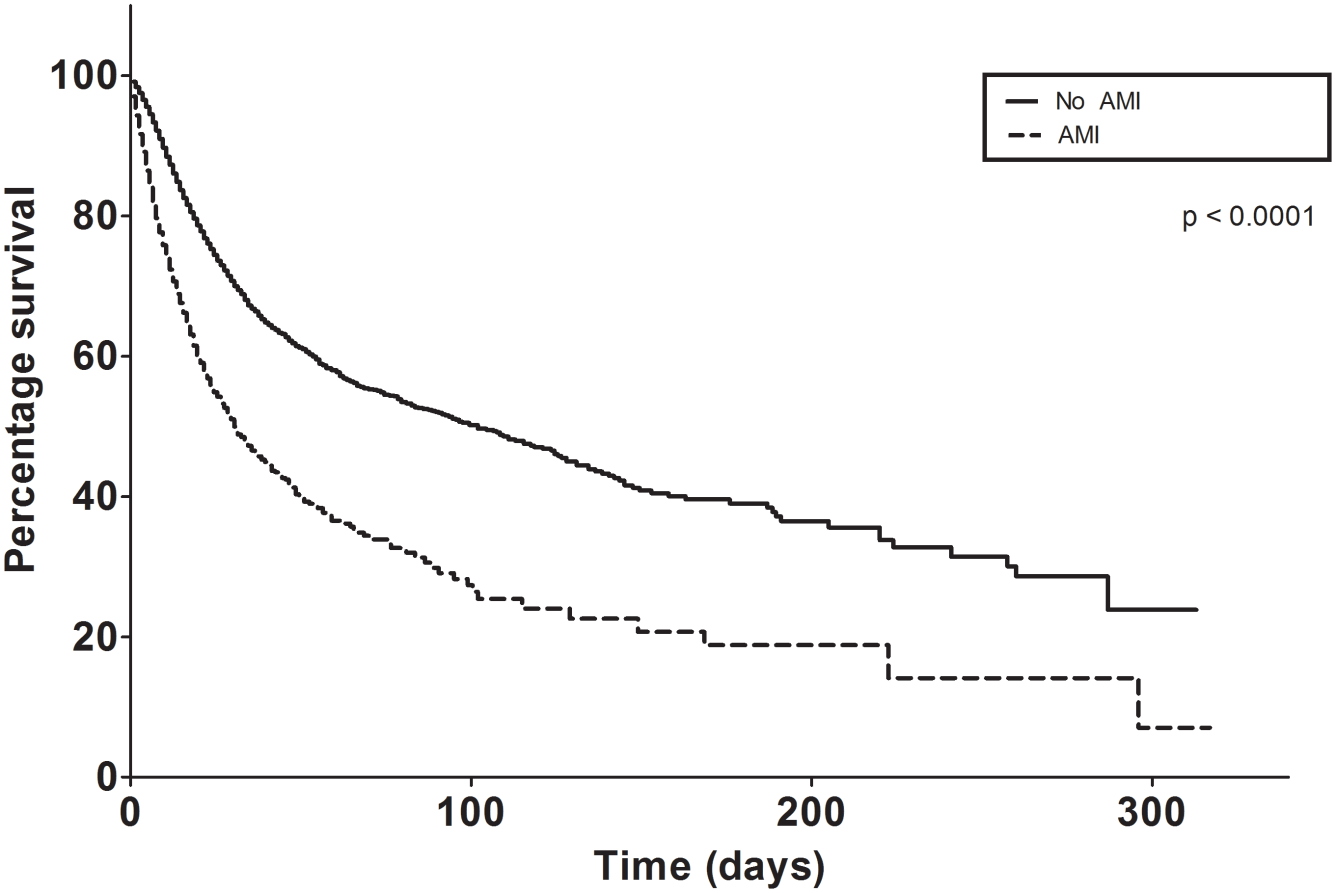
Kaplan-Meier survival analysis with and without AMI.

Our Kaplan-Meier analysis, demonstrated divergence in the survival fractions between the AMI and non-AMI groups over time for ACI patients (log-rank p<0.0001), **Figure 3**. Patients who had associated AMI had lower survival (median time to death 32 days), vs patients who had no AMI (median time to death 103 days).

## Discussion

In this retrospective analysis of primary admission diagnosis of ACI from the NIS database, our study suggests an exceedingly high mortality rate in patients who had associated AMI. However, overall mortality in this patient population has declined over the ten years studied.

During the time period under study, we noted a 1.98% rate of AMI in patients admitted with a primary diagnosis of ACI. This is analogous to previous studies with similar prevalence of AMI in their retrospective assessments. [17], [18] In a single-institutional retrospective study of in-patients with a primary diagnosis of acute stroke, Micheli and colleagues demonstrated a 1.6% incidence of acute myocardial infarction (AMI). [17] Both Micheli’s study and ours, demonstrated that the presence of AMI significantly impacted mortality during inpatient admission. Similarly, in a retrospective cohort study of the Registry of the Canadian Stroke Network, Liao and colleagues demonstrated a 2.3% incidence rate of AMI in patients admitted with ACI. [18], [19] An interesting revelation of their study was their relatively high mortality rate of 56.4% after one year. Our study showed that overall mortality in ACI is significantly continued to decline over the course of the study**.** This declining mortality may be indicative of the impact of organized inpatient stroke care, [20] and the emergence of primary stroke centers over the past decade. [21], [22] These facilities not only stabilize stroke patients in the acute period, but also focus on treating cardiovascular comorbidities that impact mortality and possibly account for the divergence in survival expectations in patients with AMI compared to those without. It must be noted that in light of a shared underlying pathophysiology in both cardiovascular and cerebrovascular diseases, it is likely that AMI may subsume the role of a severity marker in patients with diagnosed CVA. Chalela and colleagues in a retrospective assessment of acute stroke patients, demonstrated that elevated troponin concentrations correlated with stroke severity (p = 0.02) as determined by the National Institutes of Health Stroke Scale (NIHSS). [23] Their report, validated by characteristic CVA findings on MRI corroborated previously reported findings using CK-MB [24].

The academic literature has demonstrated clear associations between cerebral ischemic events and myocardial ischemia. The most common cause of death in stroke is cardiovascular disease (41%). Long-term survival after stroke may be improved by early, active, and sustained implementation of effective strategies for preventing subsequent cardiovascular events. [25] Cerebrovascular disease and many systemic vascular diseases share similar underlying vasculo-occlusive pathology and risk factors such as diabetes and hypertension. In patients with known cerebrovascular disease, there is a risk of developing comorbid occlusive vascular disease due to underlying vasculopathy (ie. coronary artery disease or peripheral vascular disease) which negatively impacts overall outcome. [26], [27], [28] Cerebrovascular events may also affect the insular cortex and increase the risk of myocardial ischemia due to autonomic dysregulation and a preceding catecholamine surge. [29] The effects secondary to ACI, and the added cardiac insults secondary to this catecholamine surge, may cumulatively increase morbidity and mortality especially in older patients. These factors may impact medical decision making including the need for reperfusion therapy [30], [31], [32].

Between 2002 and 2011, our study demonstrates an increasing report of AMI in patients hospitalized with ACI. Similarly, the risk of AMI in the subpopulation that received IV rTPA increased over the same period with a downward trend beginning in 2009**.** To date, only few studies have demonstrated the trends in cardiac events during primary admissions for cerebral ischemia.

It is possible that the increasing report of AMI events in this subpopulation is related to the growing significance of stroke admission protocols that routinely check cardiac enzymes, electrocardiogram and echocardiograms. With improved technologies and national health initiatives, cardiac events are being detected and treated at a greater rate due to increased awareness. Secondly, changes in the diagnostic criteria for myocardial infarction may have also played a role in our observations. In 2007, the *“Universal Definition of Myocardial Infarction”* was published. [33] This, consensus document, the second of its kind, revised the diagnostic criteria for AMI to delineate five classes of myocardial infarction, each differentiated by their etiologies and clinical associations. Previous recommendations validated by the European Society of Cardiology (ESC) and the American College of Cardiology (ACC), were less specific and allowed for the separate categorization of *unstable angina*, previously not characterized as myocardial infarction. [34] Through the incorporation of these recommendations in clinical practice, it may be possible that an overall increase in previously unreported myocardial injuries are now captured resulting in an increasing incidence in 2008, **Figure 1**. Subsequently, however a third iteration has been published in 2012, further revising the diagnostic criteria with greater emphasis on cardiac markers in the diagnosis [35].

We also demonstrate a significantly higher mortality rate in the subset of patients who developed AMI (22.65% vs 5.31%). This finding may be explained by a higher rate of systemic co-morbidities, **(** **Table 4** **)** and possibly higher admission NIHSS scores in these patients. Not surprisingly, survival fractions for the AMI and non-AMI group diverged significantly over time in ACI patients. The associated diagnosis of AMI was associated with decreased time to death in this patient population (mean: 103 days vs. 32 days), most likely due to co-morbidities and attendant complications.

Intravenous rTPA is the gold standard for reperfusion therapy after acute cerebral ischemia and is endorsed by the American Heart Association and the American Association of Neurology for use within three hours of sentinel infarction. [36] Notwithstanding the positive mortality benefit in this patient population, various studies have demonstrated the inherent risks of administration including the likelihood of intracerebral hemorrhages, reperfusion infarction, and secondary embolism. [37], [38] The risk of myocardial ischemia is a less studied complication that could be due to secondary embolization of pre-existing intra-cardiac or coronary emboli. [39] Small case reports suggest a low incidence and risk profile due to its rarity. [40], [41], [42] In our assessment, there was a statistically significant increase in the report of AMI in patients who received rTPA during admission compared to those who did not. Similarly, overall mortality was negatively impacted in this cohort. Causality between thrombolytic administration and the occurrence of cardio embolic disease however cannot be determined from our study, and it is also possible there may exist variability in the administration of thrombolytic based on disease severity, the presence of comorbid conditions and other variables confounding our findings. Further and more robust studies will be required to answer these questions.

In our study, ACI patients who received IV rTPA had a 6.16% mortality rate. Over the course of the study, there was a greater than two fold increase in the likelihood for inpatient mortality in this subset, compared to those who did not require thrombolysis, **Table 5** **.** This estimate of inpatient mortality in this treatment group is concordant with previously reported estimates in similar studies. Reed and colleagues in their retrospective assessment of patients who received IV rTPA in the treatment of ischemic strokes demonstrated an inpatient mortality rate of 9.9% (95% CI 6.9%–13.0%). [43] One interesting point in their study was the large variability in administration based on multiple factors including race, age and gender. Additionally, in a large multi-institutional study, researchers in Germany demonstrated 10% mortality in patients receiving IV rTPA therapy, [44] with increasing mortality risk associated with increasing age. As expected, congestive heart failure was also associated with increased odds of death in this cohort, however hypertension, and diabetes mellitus, both pathologies associated with vasculopathy did not demonstrate similar trends, **Table 5**. These observations are not readily explained and will require more rigorous studies.

**Table 5:** Multivariate analysis predicting the odds of mortality.

Comorbidities	Odds Ratio	95% Confidence Interval	P value
Congestive Heart Failure	1.94	1.89–1.99	0
Alcohol use	1.08	1.01–1.15	0.022567
Coagulopathy	1.75	1.66–1.85	<0.0001
Diabetes	0.97	0.94–0.99	0.015118
rtPA	2.39	2.11–2.71	<0.0001
Myocardial Infarction	3.68	3.49–3.88	0
Obesity	0.83	0.78–0.88	<0.0001
Hypertension	0.62	0.61–0.64	0

### Limitations

This study has limitations that implicitly affect its interpretation. A major limitation to our investigation is the definition of AMI used. Since this study is based on ICD-9 codes for diagnosis of AMI, the diagnostic tools used by physicians involved with these stroke patients is unclear. This is a general limitation of NIS database but other clinical studies have relied on the rates of myocardial infarction reported in NIS. [45] The initial diagnosis of ACI and AMI is dependent on either clinical suspicion or standardized protocols. Therefore, practice variability throughout this study inevitably affects the likelihood of diagnosis and reporting. Additionally, as awareness of AMI in stroke patients increases, a general increase in clinical suspicion occurs and thus, increases its reporting rates. Similar to prior studies, [46] our analysis was limited by its observational design, and therefore, etiological or causal relationships between variables and outcomes cannot be entirely assumed. ICD-9-CM codes have historically been of questionable accuracy, particularly since they may change over time. We may have also potentially missed cases of AMI not evaluated at an acute care hospital. Myocardial infarction has multiple etiologies and various diagnostic code associations. The focus of this study was on those etiologies that may present with ischemic and hypoxic sequelae as presented by ICD-9 codes 410.0 through 410.9. While sub-endocardial infarction may not present with EKG changes, and may not impact mortality to the same extent as non-segmental infarction, the mutual pathophysiology and risk factors makes it a vital component of our analysis. Information regarding comorbidities and other covariates affecting morbidity and mortality after stroke is not represented in this database, precluding the ability to assess their association to morbidity and mortality in this observational study. Data on the disposition of patients at discharge may have been influenced by the overall increase in the use and transfers to long-term health care facilities in the United States predicated on the recent changes in insurance reimbursement and hospital overcrowding. We were also unable to examine or link mortality data beyond the hospital admission. Of other variables that may have confounded our observed associations such as the time of onset of ACI, additional comorbidities, severity and characteristics of ACI, rates of DNR orders, and timing of treatments.

### Conclusion

Although AMI is uncommon in ACI patients, but it independently predicts inpatient mortality and survival. Additionally the administration of IV rTPA is associated with an increased report of AMI and mortality, but it is decreasing during recent years.

Considering the high clinical burden of AMI on mortality of ACI patients, a high quality monitoring care for suspicion of cardiac events should be maintained in these particular patients. Robust and meticulous care should be exercised in the management of these patients given their propensity for rapid clinical decline and high rate of mortality. Whether a prompt diagnosis and treatment of associated cardiovascular diseases, may improve outcome will remain to be explored in appropriate clinical studies.
